# Modeling dyslexia in neurotypical adults by combining neuroimaging and neuromodulation techniques: a hypothesis paper

**DOI:** 10.3389/fnhum.2025.1651332

**Published:** 2025-10-10

**Authors:** Daniel Gallagher, Zian Huang, Shinri Ohta

**Affiliations:** 1 Department of Linguistics, Faculty of Humanities, Kyushu University, Fukuoka, Japan; 2 Department of Linguistics, Graduate School of Humanities, Kyushu University, Fukuoka, Japan

**Keywords:** dyslexia, dyslexia subtypes, human models, functional magnetic resonance imaging (fMRI), neuropathological clustering, neuromodulation, non-invasive brain stimulation (NIBS), transcranial temporal interference stimulation (tTIS)

## Abstract

Dyslexia is a prevalent developmental disorder marked by deficits in literacy skills. Given that the core deficits of dyslexia are uniquely human, animal models have not been as useful in dyslexia research as they have been in other areas of research. While significant progress has been made through behavioral and neuroimaging studies, a viable model could facilitate controlled investigations into the neural mechanisms underlying dyslexia and accelerate the development of targeted interventions. In this hypothesis article, we propose a two-pronged approach to model dyslexia in neurotypical adults using neuroimaging and neuromodulation techniques. First, we propose using functional and structural MRI data to cluster individuals into neuropathologically derived subgroups in order to facilitate the classification of dyslexia subtypes based on neuropathological characteristics. Second, we propose employing transcranial temporal interference stimulation (tTIS) to temporarily downregulate activity in brain regions specified in the clustering analysis, inducing subtype-specific dyslexic symptoms in neurotypical individuals. This approach enables the establishment of causal or probabilistic relationships between neuropathologies and dyslexia subtypes, while at the same time creating dyslexia models to facilitate investigation into subtype-specific interventions. Although this model is somewhat limited by the transient nature of neuromodulation as well as by the use of healthy adults to model a developmental disorder whose symptoms first arise in childhood, it is a meaningful step towards refining our understanding of the neural basis of dyslexia subtypes and it opens the door to novel and effective therapies. By integrating neuroimaging and neuromodulation, we hope to offer a viable substitute for animal models in dyslexia and accelerate the development of personalized therapeutic strategies for dyslexia.

## 1 Introduction

As a learning disability causing literacy skill deficits, dyslexia, which includes several subtypes, is one of the most prevalent developmental disorders affecting the human population, affecting about 7% of the population (Yang et al., 2022). Broadly, subtypes of dyslexia have been identified based on impairments in phonological awareness (PA), rapid automatized naming (RAN), and visuo-spatial processing, among other cognitive domains (Bruck, 1992; Denckla and Rudel, 1976). As the heterogeneity of dyslexia has come into greater focus, models have gradually shifted from emphasizing single deficits to double deficits to multiple deficits (Pennington, 2006; Wolf and Bowers, 1999). Although the multiple deficit model provides a strong explanatory framework (McGrath et al., 2020; Pennington, 2006), the most appropriate approach to classifying dyslexia subtypes remains an open debate.

Some neuroimaging research has provided significant insights into the neurobiological basis of dyslexia subtypes, highlighting structural and functional differences in key reading-related brain regions. For example, hypoactivation, atypical connectivity patterns, and structural variations in gray matter volume have all been reported to vary according to subtype (Jednoróg et al., 2014; Norton et al., 2015). Despite these advances, most studies investigating the neural mechanisms of dyslexia do not account for specific behavioral deficits, representing a gap in the dyslexia literature.

In response to this gap and in light of recent technological developments, we propose a two-part hypothesis for the investigation of dyslexia subtypes:

1. Developmental dyslexia leads to subtype-specific anatomical and functional abnormalities in the brain, and
2. Transcranial temporal interference stimulation (tTIS) can induce targeted functional deficiencies in the brain and elicit subtype-specific models of dyslexia.

Progress towards developing and testing new treatments is further hindered by the heterogeneity of the disorder, which makes it challenging to recruit sufficiently large and homogenous participant groups. In other neurodevelopmental and neurological disorders, such as attention-deficit hyperactivity disorder (ADHD), autism, etc., this challenge is often mitigated by utilizing appropriate animal models (Russell, 2011; Varghese et al., 2017), which replicate the key symptomatic expressions of the disorder being studied. For example, rats may be selectively bred to achieve specific symptoms, as is done with spontaneously hypertensive rats in ADHD research (Sagvolden et al., 1992). In other cases, the genes of a mouse are modified (e.g., knockout and knockin mice), and still in other cases, drugs may be administered to induce the desired symptoms. Although not without their limitations (Robinson et al., 2019), animal models serve as an important investigative tool that allows researchers to delineate neuropathologies and assess the viability of various therapeutic and pharmacological interventions (Mukherjee et al., 2022). These models allow researchers to do this substantially faster than would otherwise be possible solely using human subjects, ultimately shortening the time it takes for novel interventions to reach clinical implementation.

However, unlike other neurological disorders, dyslexia specifically affects literacy skills, which are uniquely human. Therefore, an animal model of dyslexia cannot adequately replicate the challenges faced by human individuals with dyslexia. Even if, for example through genetic modification, “dyslexia” were to be artificially induced in an animal, it is unclear how it should manifest and what symptoms should present. Nevertheless, the approach has been taken in genetic studies of dyslexia. In one study, KIAA0319 knockdown rats—where the KIAA0319 gene was suppressed by RNA interference—presented with impaired phoneme processing in the primary auditory cortex (Centanni et al., 2014). In another genetic study, DCDC2 knockdown rats presented with speech sound discrimination deficiencies (Centanni et al., 2016). These and similar studies offer crucial insights into the multifactorial genetic underpinnings of dyslexia and are indeed important pieces of the puzzle. However, as intriguing as these studies are, perfect translation to humans cannot be assumed. Consider, for example, FOXP2, which is often heralded as a key gene in speech and language development (Lai et al., 2001). Yet homologues are conserved across many distant species, including those with vocal learning systems, such as songbirds, and those entirely without, such as fruit flies, where the roles of these genes diverge substantially from their function in humans (Castells-Nobau et al., 2019; Hannenhalli and Kaestner, 2009; Lai et al., 2001). This illustrates that the function of a gene can vary dramatically depending on species-specific regulatory networks, developmental pathways, and environmental contexts. Ultimately, because animals are incapable of literacy, it is not possible to surmise whether the induced symptoms truly reflect dyslexia or just peripherally related symptoms, and by extension therefore, it is not possible to assess the extent of applicability of the models to human dyslexia.

Dyslexia researchers are therefore faced with a unique challenge, where they must either rely on genetically modified animals with an unknowable degree of symptomatic specificity or work exclusively with dyslexic individuals, which necessarily entails higher hurdles related to recruiting, turnaround times, interindividual differences, etc. (Roitsch and Watson, 2019). Thus, a reliable model of dyslexia would fill a gap in investigatory approaches in dyslexia research. However, again, since literacy skills—the principal marker of dyslexia—are unique to humans, an animal model is simply not feasible. We therefore propose the development of a dyslexia model in neurotypical adult humans.

As mentioned, the successful creation of this dyslexia model hinges on a two-part hypothesis that dyslexia subtypes lead to differentiated neural anomalies and that we can temporarily induce the functional anomalies with tTIS. Correspondingly, we take a two-pronged approach to developing the model. In the first prong, we establish the neurological basis of dyslexia by analyzing open-source brain data on adults and children with and without dyslexia in order to elucidate the most relevant brain abnormalities associated with the disorder and select target regions. In the second prong, we employ tTIS, which can achieve both focal and deep brain stimulation without stimulating surrounding areas, at the target regions specified by the structural and functional analysis to induce subtype-specific dyslexic symptoms in neurotypical adults. In other words, we expect that by stimulating a given brain region associated with a given subtype of dyslexia, only the symptoms of that subtype should be elicited. In this way, subtype-specific dyslexia models can be created in neurotypical adults, facilitating the development of more targeted and individualized interventions for treating dyslexia.

## 2 Background

### 2.1 Neural mechanisms of typical Reading and dyslexia

Dyslexia in the broad sense is behaviorally characterized by a deficiency in literacy skills, such as lower reading accuracy or fluency, without affecting general intelligence or other linguistic abilities. The most prominent region employed in the reading network is well-known to be the left fusiform gyrus (FG), also known as the visual word form area (VWFA). For example, it has been shown that reading speeds positively correlate with the degree of VWFA activation (Christodoulou et al., 2014; Langer et al., 2015). More broadly, however, the cognitive act of reading consists of both cooperative and competitive mechanisms recruiting many areas involved in orthographic, phonological, and semantic processing, such as the left inferior frontal gyrus (LIFG) and left temporal, left inferior parietal, and occipito-temporal regions (Cattinelli et al., 2013).

In typical readers, the classical pattern of activation is broadly segregated into two pathways: the dorsal and ventral pathways, which align with reading by decoding and sight reading, respectively. When a typical reader decodes a word, letters are mapped onto their pronunciations, and the whole-word pronunciation is constructed in a phonological, bottom-up process. This process activates the dorsal pathway, which includes the left inferior frontal gyrus (Broca’s area), precentral gyrus, and temporo-parietal regions (e.g., perisylvian regions and Wernicke’s area). When sight reading a word, the word is recognized as a whole, drawing on lexical knowledge and context to access the word in a less phonological, more top-down approach. This process activates the ventral pathway, which includes the left inferior frontal gyrus and occipito-temporal cortex (including the VWFA). It is also noted that subcortical structures such as the striatum and thalamus play a less-understood role in reading (Kearns et al., 2019).

Dyslexia is marked by both structural and functional abnormalities in the regions of this typical reading network as well as compensatory activations elsewhere. For example, using fMRI, Shaywitz et al. revealed a functional disruption in the reading network of individuals with dyslexia characterized by hypoactivation of dorsal pathway regions, including the superior temporal gyrus (Wernicke’s area) and the angular gyrus (Shaywitz et al., 1998). Similarly, the ventral pathway has also been shown to have disrupted functional connectivity in the VWFA of individuals with dyslexia (Brem et al., 2020; Van der Mark et al., 2011). Finally, it is regularly observed that in adults with dyslexia, other brain regions are recruited to help compensate for the dysfunction of the typical reading network. For example, hyperactivation is observed in the left inferior frontal gyrus (Broca’s area), as well as in right posterior regions (Pugh et al., 2000). This hyperactivation is often interpreted as a compensatory mechanism for left hemisphere posterior hypoactivation. Building on these hypo- and hyperactivations, the neural noise hypothesis of developmental dyslexia proposes that dyslexia arises from variability or ‘neural noise’ within these regions of the reading network (Hancock et al., 2017). This hypothesis has found some supporting evidence in an fMRI study conducted by Malins and colleagues, underscoring the heterogeneous nature of developmental dyslexia (Malins et al., 2018).

On the structural side, a meta-analysis of voxel-based morphometry (VBM) studies showed that individuals with dyslexia exhibited gray matter reduction in the right superior temporal gyrus and left superior temporal sulcus, while the VWFA was shown by several individual studies to have gray matter reduction without meeting clustering criteria for the meta-analysis (Richlan et al., 2013). Another meta-analysis focusing on functional abnormalities revealed consistent hypoactivation in the left inferior parietal lobule, LIFG, and superior, middle, and inferior temporal regions, and fusiform regions (e.g., VWFA), as well as hyperactivation of the primary motor cortex and anterior insula (Richlan et al., 2009). However, as a neurodevelopmental disorder, it makes intuitive sense that dyslexia should affect individuals differently at different stages of development. Indeed, when controlling for age group, the results diverged slightly. It was found that while both children and adults with dyslexia exhibited hypoactivation in the left ventral occipital-temporal region (which includes the VWFA), only children exhibited hypoactivation in bilateral inferior parietal regions, while only adults showed hypoactivation in the superior temporal regions (Richlan et al., 2011).

### 2.2 Dyslexia subtypes

These findings have already clarified a great deal of the neurobiology of dyslexia. Nevertheless, most neuroimaging studies of dyslexia are confounded by the heterogeneity of the disorder, which can be partially alleviated by appropriately classifying individuals according to their specific deficit(s). Thus, it is necessary to identify the distinctive neural bases of the different dyslexia subtypes. To that end, Norton et al. (2015) neatly summarized the contemporary understanding of the brain bases of behaviorally derived phenological subtypes of dyslexia. Based on their findings, some core behavioral deficits associated with dyslexia are recognized: Phonological awareness (PA), rapid automatized naming (RAN), and sensory and working memory related processes (Norton et al., 2015).

In the phonological deficit hypothesis of dyslexia, it is thought that dyslexia is the result of poor phonological skills hindering the acquisition of the rules governing spelling (Snowling, 1998). These PA deficits manifest in behavioral experiments as impaired repetition and decoding of nonwords (Rack et al., 1992; Snowling, 1981) and impaired recognition of rhymes and alliterations (Bradley and Bryant, 1978). Such deficits have been shown to arise from functional and structural connectivity (as measured by diffusion tensor imaging) between auditory cortices and the LIFG, reduced prefrontal activation, but no abnormalities in temporal lobe activation (Boets et al., 2013).

In RAN deficits, individuals with dyslexia exhibit markedly slower naming speeds for colors, numbers, letters, and objects (Denckla and Rudel, 1976). These deficits are less localized than PA deficits and are associated with more whole-brain volumetric differences (He et al., 2013), as well as lower activation in the right cerebellar lobule VI (Norton et al., 2014).

Some studies have also shown that individuals with dyslexia may have various abnormalities in sensory and working memory related processes, such as reduced left-lateralized entrainment at frequencies critical for parsing speech signals (Giraud and Poeppel, 2012; Lehongre et al., 2013), reduced left-lateralized integration of phonological and orthographic information (Hasko et al., 2012), and reduced bilateral activation in the BA7 leading to a working memory deficit related to temporal order processing (Beneventi et al., 2009).

In one study specifically comparing various dyslexia subtypes, Jednoróg et al. (2014) categorized dyslexic children into subtypes based on behavioral assessments, including PA, RAN, and sensory deficits, and found specific gray matter patterns aligning with the dyslexia subtypes. Specifically, their voxel-based morphometry (VBM) approach revealed the LIFG, cerebellum, right putamen, and bilateral parietal cortex as areas with gray matter volume differences between the different dyslexic subtypes (Jednoróg et al., 2014). To our knowledge, the functional distinctions between subtypes have been less thoroughly investigated. Nevertheless, a 2013 study that compared non-phonological dyslexics to phonological dyslexics exhibited heightened activation in several key areas, including the left inferior frontal gyrus, supplementary motor area, and precentral gyrus, as well as the right insula (Van Ermingen-Marbach et al., 2013). They also showed that non-phonological dyslexics exhibited heightened activation in the left supramarginal and angular gyri. In either group, various hyperactivations aligned with regions employed in the dorsal pathway of reading, while none aligned with the ventral pathway.

While some studies have indeed shown dyslexia subtype-specific functional abnormalities, further research is needed to gain a more comprehensive understanding of dyslexia subtypes. For example, rather than assuming *a priori* that dyslexic participants can and should be grouped by type of behavioral deficiency, we propose that beginning with neuropathologically-derived clusters may elucidate heretofore unobserved patterns in dyslexia.

### 2.3 The multiple deficit model of developmental disorders

Thus far, we have discussed dyslexia as though there is a one-to-one correspondence between a given brain anomaly and a specific behavioral symptom. However, the multiple deficit model (MDM) has gained prominence in recent years and challenges this notion by proposing that developmental disorders, including dyslexia, arise from the interactive effects of multiple risk factors rather than a single causal deficit (Pennington, 2006). Unlike single-deficit and double-deficit models, which assume that a particular brain anomaly leads directly to a specific cognitive impairment, the MDM conceptualizes dyslexia as a probabilistic outcome resulting from the accumulation and interaction of multiple neural, genetic, and environmental influences.

From this perspective, a given brain anomaly does not necessarily and deterministically produce a specific deficit but rather increases the probability of it. Conversely, the same cognitive symptom can arise from different underlying neural anomalies in different individuals. This model has demonstrated more reliable predictive power than single-deficit models, particularly for individuals with dyslexia and dyscalculia, though a hybrid approach using the different models in tandem seems to outperform using either model exclusively (McGrath et al., 2020; Pennington et al., 2012). Importantly, within this framework, the discussion of deficits and subtypes turns from “causally deterministic” to “probabilistically predictive,” and rather than speaking of “core deficits” of dyslexia (such as PA and RAN deficits), the more appropriate terminology is “predictors.” By removing *a priori* assumptions that a given neural abnormality must lead to a specific outcome, this shift in perspective effectively accounts for the heterogeneity of dyslexia, bridging behavioral variability with multifaceted neuropathologies. At first glance, this seems to undermine our hypothesis that neuromodulation can reliably induce specific dyslexia symptoms. However, rather than invalidating our approach, MDM simply alters the framing of the approach. That is, by inducing hypoactivation in a given brain region(s) implicated in dyslexia, we expect with some degree of likelihood that an associated symptom(s) will present. In other words, we simply shift away from deterministic one-to-one mappings and towards probabilistic many-to-many mappings.

Thus, even assuming the MDM framework and discarding the notion of deterministic relationships between specific brain regions and symptoms, studying the likelihood of specific neural anomalies contributing to particular cognitive deficits remains crucial for understanding the neurobiology of dyslexia and guiding the development of effective interventions. By identifying which brain regions are most likely to contribute to specific deficits, we can refine dyslexia diagnosis and develop more targeted therapies that address the individualized constellation of risk factors present in each case.

### 2.4 Defining the neurological pathogenesis of dyslexia subtypes

Of the neuroscientific studies targeting dyslexia subtypes, most have started with the symptomatic manifestations of dyslexia and then aimed to elucidate the neural patterns associated with those symptoms. However, due to the complex etiology of dyslexia, it is challenging to fully describe the neural basis of specific dyslexia subtypes using only monomodal analyses. To solve this challenge, we propose grouping individuals with dyslexia based on similarities in their structural and functional MRI data in order to uncover novel, neuropathologically defined subtypes.

Multimodal neuroimaging has been used extensively and in various ways to investigate dyslexia. For example, Hoeft and colleagues combined children’s structural brain data, functional brain data, and behavioral performance at the beginning of the school year in order to predict letter decoding skills at the end of the school year. Both brain data alone and combined with earlier behavioral measures accurately modeled decoding skill trajectories for children with dyslexia (Hoeft et al., 2007). Other recent approaches have involved describing comorbidities of dyslexia, combining electroencephalography with MRI, and even using deep-learning approaches for dyslexia detection (Alkhurayyif and Sait, 2024; Hernández-Vásquez et al., 2023; Nemmi et al., 2023). However, to the best of our knowledge, multimodal neuroimaging has not yet been combined with neuropathological clustering to describe subtypes of dyslexia, despite previous research suggesting that neuroimaging may provide greater predictive power on later clinical outcomes than behavioral measures (Norton et al., 2015). Thus, our proposal to do so represents a distinct perspective, contrasting with previous research approaches that focus on subtyping dyslexia by specific behavioral patterns or symptomatic manifestations. By emphasizing the identification of subtypes through structural and functional brain data, we expect to provide new insights for targeted interventions tailored to each subtype of dyslexia.

By combining this approach with tTIS, we suggest that the relationships between the implicated brain regions and specific dyslexia symptoms can be established, and dyslexia models can be created in neurotypical adults. Previous studies employing other forms of non-invasive brain stimulation (NIBS), such as transcranial direct current stimulation (tDCS) and transcranial magnetic stimulation (TMS), have already demonstrated stimulation at various sites as an effective therapeutic intervention regardless of age group, though the pressing need for more NIBS studies is acknowledged (Turker and Hartwigsen, 2022). Towards that goal, tTIS in particular can fill an important gap in the dyslexia-NIBS literature thanks to its unique capability to stimulate previously inaccessible regions, including the VWFA.

The aforementioned dysfunction observed in the VWFA, LIFG, cerebellum (lobule VI), and superior temporal regions suggests the viability of targeting these regions for localized brain stimulation in adults. However, by additionally looking at clusters of neuropathologies found in individuals with dyslexia, other regions of interest may be observed and subsequently tested via tTIS. In the subsequent sections, we will describe the specific methodologies and benefits of our two-pronged approach to developing a dyslexia model in neurotypical adults.

## 3 Functional and structural MRI analysis

The first prong of our approach aims to identify the brain regions associated with dyslexia subtypes by employing two complementary methods to assess brain anomalies: structural magnetic resonance imaging (MRI) to examine brain anatomy and functional MRI (fMRI).

By analyzing both structural and functional data, it is possible to determine how structural changes correlate with patterns of functional connectivity. This could lead to a more nuanced understanding of dyslexia and provide valuable information for intervention strategies. Future avenues can explore more detailed functional connectivity analysis or the use of other brain atlases and ROIs to refine our understanding of the relationship between brain structure and function in dyslexia. Importantly, identifying converging structural–functional abnormalities also provides candidate target regions for transcranial temporal interference stimulation (tTIS), enabling us not only to map the neural basis of dyslexia but also to directly inform stimulation-based experiments and interventions.

### 3.1 Structural MRI analysis

By performing voxel-based morphometry (VBM) and surface-based morphometry (SBM), it is possible to elucidate significant structural differences between individuals with dyslexia and controls. VBM analysis focuses on the 3D volume of brain tissue and is primarily used for analyzing gray and white matter volumes across brain regions; while SBM works with the cortical surface, analyzing features such as cortical thickness, sulcal depth, and gyrification, and is often used to study more localized cortical regions (Goto et al., 2022).

Through VBM, it is possible to assess whether dyslexic adults exhibit reductions in grey/white matter volume in regions crucial for language processing and visual–spatial integration. For instance, reductions in the volume of key white matter tracts, such as the arcuate fasciculus (Žarić et al., 2018) and the inferior fronto-occipital fasciculus (Lou et al., 2019), may indicate decreased efficiency in neural connectivity, potentially reflecting challenges in recruiting neural resources. Additionally, reductions in the left corpus callosum may disrupt interhemispheric communication, impacting functions commonly associated with the right hemisphere (Hynd et al., 1995).

Moreover, SBM analysis uniquely allows for the measurement of cortical thickness, enabling the detection of subtle structural changes that may not be captured by other voxel-based methods. Through SBM, cortical thinning can be identified in key regions, such as the left fusiform gyrus (FG), superior temporal gyrus (STG), middle temporal gyrus (MTG), and inferior frontal gyrus (IFG).

While VBM and SBM work as whole-brain analyses requiring correcting for thousands of voxels/vertices (e.g., FWE or FDR correction), making it harder to find significant effects, ROI-based analysis limits comparisons to a smaller number of voxels/vertices, which lowers the correction burden. However, it also relies on prior knowledge to define ROIs, which may introduce bias and limit the discovery of unexpected findings. Combining whole-brain and ROI-based approaches can thus provide a more comprehensive understanding of the structural alterations associated with dyslexia (Poldrack, 2007).

To mitigate potential biases introduced by ROI-based analysis, we will additionally employ data-driven approaches, including source-based morphometry using ICA (Xu et al., 2009) and structural covariance/morphometric similarity network methods (Alexander-Bloch et al., 2013; Seidlitz et al., 2018). These approaches allow the delineation of structural alterations without relying on *a priori* ROI definitions, thus reducing bias and increasing reproducibility.

Notably, cortical thinning in these regions might be more pronounced in older adults with dyslexia, potentially reflecting an age-related pattern that offers valuable insights into the evolution of dyslexia across the lifespan. Furthermore, it is promising to investigate whether there might be differential aging patterns between the left and right hemispheres, as compensatory mechanisms could be at play in dyslexic brains. These findings could help guide the selection of optimal stimulation sites for targeted interventions aimed at enhancing cognitive functions related to dyslexia.

Preliminary analyses (see Supplementary file) were conducted using data obtained from OpenNeuro, specifically from Banfi et al. (2022) (originally published as Banfi et al. (2021)) and Cavalli et al. (2023). These analyses revealed significant structural differences between dyslexic adults and controls (corrected *p* < 0.05) (Supplementary Table 1). Dyslexic adults exhibited widespread white matter reductions, particularly in the left corpus callosum, and reductions in key tracts such as the left arcuate fasciculus and right inferior fronto-occipital fasciculus. Cortical thinning was observed in the right FG and rostral anterior cingulate cortex (rACC), with an aging-related pattern showing greater thinning in dyslexic adults compared to controls (Supplementary Figures 1, 2). These findings suggest that structural deficits in dyslexia extend beyond the left hemisphere, possibly reflecting compensatory mechanisms, and may impact connectivity and cognitive functions.

However, since brain stimulation can modulate neural activity, relying solely on structural results does not offer a robust or reliable foundation for defining stimulation targets. Therefore, in our future analyses, integrating both structural and functional MRI findings to identify areas of overlap between the two will yield more meaningful insights.

### 3.2 Functional MRI analysis

To investigate the neural mechanisms underlying dyslexia, we hypothesize that functional differences between individuals with dyslexia and typical readers are associated with structural brain alterations in dyslexic individuals. To test this hypothesis, we will employ a comprehensive analysis of both functional and structural MRI data.

Given that the research of both data sets focuses on the functional activity differences between the dyslexic group and typical readers, we expect to first replicate findings from previous studies to confirm the existence of functional differences. Additionally, by integrating the observed structural difference in dyslexic brains with the distinct functional patterns associated with dyslexia, it is possible to further explore if their functional patterns contribute to or reflect structural changes. If structural and functional findings do not align, we will prioritize regions showing multimodal convergence while also considering targets identified in structural-only or functional-only analyses, as such discrepancies may reflect compensatory or developmental differences.

Secondly, to effectively investigate the efficacy of dyslexia interventions, it is crucial to gain a comprehensive understanding of the brain networks and pathologies underlying the condition. Previous research has shown that disrupted network interactions serve as a neural marker for dyslexia, with dyslexic individuals exhibiting abnormal task-related functional connectivity that negatively impacts reading performance (Turker et al., 2023). To investigate dyslexia-related network changes, fMRI data will be preprocessed, and potential confounds such as motion artifacts, scanner noise, and task compliance will be carefully monitored and controlled. Using generalized psychophysiological interactions (gPPI), it is possible to characterize task-related modulation to reveal specific changes in whole-brain connectivity between subject groups (McLaren et al., 2012). Additionally, by computing ROI-to-ROI connectivity (RRC) as outlined by Nieto-Castanon and Whitfield-Gabrieli (2022), we can examine functional connectivity between regions of interest (ROIs) and investigate how these patterns differ between dyslexic and typical readers. Previous research has shown that disrupted network interactions serve as a neural marker for dyslexia, with dyslexic individuals exhibiting abnormal task-related functional connectivity that negatively impacts reading performance (Turker et al., 2023).

Through this methodology, we aim to clarify how functional connectivity relates to structural alterations in key brain regions. If any regions show alignment between functional and structural results, they will be promising targets for transcranial temporal interference stimulation (tTIS). Regions exhibiting both structural alterations (e.g., reduced cortical thickness or volume) and functional connectivity disruptions may indicate core neural deficits, while those identified in only one modality may reflect compensatory or complementary processes. These areas are likely to play a crucial role in reading-related processing, making them potential candidates for targeted neuromodulation.

### 3.3 Principal component analysis

The variability observed in our preliminary results of structural data (see Supplementary materials) still showed a larger variance than controls, reflecting the heterogeneous nature of dyslexia and suggesting the possibility that the data sets combined different subtypes of dyslexia. Therefore, we plan to apply principal component analysis (PCA) to reduce the dimensionality of structural and functional MRI data and identify the principal components that best explain differences between dyslexic subtypes. PCA simplifies complex data by extracting the majority of variance (see, e.g., Stoyanov et al., 2019).

We first propose that PCA can be used to identify brain regions with the most significant structural deviations in characteristics such as cortical thickness, volume, and density. These deviations may correspond to distinct brain structures associated with dyslexia subtypes, independent of symptoms. Rather than categorizing participants by pre-defined subtypes, PCA will group participants based on structural anomalies revealed by the data itself, offering a data-driven approach. To enhance the clarity, Varimax rotation will be applied, so that each brain region strongly loads onto only one (or a small number of) component(s), thereby revealing clearer structural patterns (Van Boxtel, 1998). Factor loadings will show how each component contributes to structural variability, while factor scores will reflect how these components relate to individual participants. These scores can be analyzed to see if structural differences vary systematically between dyslexic and control groups, providing insight into potential neuroanatomical subtypes of dyslexia. Here, Varimax is preferred for structural data because it produces orthogonal (uncorrelated) components, which enhances interpretability of distinct brain regions. While oblique rotations allow correlated components, they can make structural interpretation more complex, so we use Varimax as a first exploratory step.

Beyond structural analysis, PCA can be extended to functional connectivity data to explore whether the identified subgroups also exhibit distinct functional connectivity patterns. In this case, oblique rotations (e.g., Promax or Oblimin) are suitable, as they allow correlated components that better reflect the interrelated nature of reading-related brain networks (Abdi, 2003). PCA applied to the functional connectivity matrix can thus extract principal components that capture variance associated with potential dyslexia subtypes, enabling detection of functionally meaningful subnetworks while maintaining interpretability of connectivity patterns.

To ensure the robustness of PCA-derived subtypes, we propose incorporating a train/test approach. Specifically, the dataset will be split into a training set for initial PCA extraction and a testing set to confirm the stability of the identified components. Cross-validation techniques (e.g., k-fold validation) will further prevent overfitting and evaluate reproducibility across datasets (Banfi et al., 2022; Cavalli et al., 2023). Extracted factors can also be applied to independent datasets to confirm reliability.

These subtype-specific patterns form the foundation of neuroscientific diagnostics by linking structural and functional data to functional impairments, ultimately contributing to more personalized and targeted interventions.

## 4 Transcranial temporal interference stimulation (tTIS) for modeling dyslexia subtypes

The second prong of our approach involves using tTIS to stimulate the various regions highlighted by the functional and structural analysis.

### 4.1 Non-invasive brain stimulation (NIBS)

Multiple NIBS methods have shown great utility in cognitive neuroscientific studies, especially for establishing causal relationships between brain regions and cognitive functions (Bergmann and Hartwigsen, 2021) and for providing a novel and promising therapeutic intervention for disorders like autism and dyslexia (Lazzaro et al., 2022; Sokhadze et al., 2014; Turker and Hartwigsen, 2022). For example, tDCS has revealed the causal role of the LIFG in second-language grammar acquisition (Gallagher et al., 2022), the causal role of the temporo-parietal cortex in novel word learning (Perceval et al., 2017), and the domain-specificity of the dorsolateral prefrontal cortex in bilingual language control (Vaughn et al., 2021). Transcranial alternating current stimulation (tACS) has also revealed important relationships, such as theta-phase synchronization in the frontoparietal regions causing visual memory matching (Polanía et al., 2012), neural entrainment to speech causing intelligibility (Riecke et al., 2018), and gamma activity in the temporal lobe causing moments of insight (Santarnecchi et al., 2019). TMS has also been utilized to delineate functionally distinct language regions in the brain as well as to enhance performance in a variety of cognitive domains such as picture naming, numerical discrimination, and word recognition (Devlin and Watkins, 2007; Luber and Lisanby, 2014).

In dyslexia, three recent (systematic) reviews have already concluded that NIBS techniques are a promising remedial tool for reading deficits across age groups and languages, particularly emphasizing the efficacy of tDCS over the left temporo-parietal cortex (Cancer and Antonietti, 2018; Salehinejad et al., 2022; Turker and Hartwigsen, 2022). For example, several studies showed that stimulation (tDCS, TMS) over left temporo-parietal regions improved reading performance. Using TMS over the left superior temporal gyrus, Costanzo et al. were able to increase word reading speed and text reading accuracy (Costanzo et al., 2013). And the same group using TMS over the inferior parietal lobule was able to improve pseudoword decoding (Costanzo et al., 2013). Stimulation of the left posterior temporal cortex resulted in improved reading efficiency in below-average readers (Turkeltaub et al., 2012). And anodal tDCS over the middle visual field improved reading speed and fluency in a RAN-based task (Heth and Lavidor, 2015).

Importantly, tDCS has also been shown to have long-lasting benefits when used over several sessions. For example, Costanzo et al. showed that anodal and cathodal tDCS over the left and right temporal parietal junctions, respectively, coupled with training resulted in long-lasting pseudoword and text reading benefits (Costanzo et al., 2019). More recently, Rezaei and colleagues stimulated the left temporo-parietal region with anodal tDCS over 12 sessions, also showing long-lasting improvements in low-frequency word reading and nonword decoding (Rezaei et al., 2025). This demonstration of lasting effects is crucial for the meaningful use of tDCS as a therapeutic intervention.

Compared to tDCS, tACS studies are substantially underrepresented in the dyslexia literature. Nevertheless, tACS has also proven effective at enhancing reading skills with a unique advantage. Because tACS delivers alternating current, it can be tuned to oscillate at frequencies that naturally align with optimal processing rhythms in the brain. In particular, gamma range stimulation (e.g., 30–40 Hz) over the auditory cortex has been shown to be effective for improving phonemic/phonological awareness and phonemic categorization (Marchesotti et al., 2020; Rufener et al., 2019; Rufener and Zaehle, 2021).

Thus, it has already been reliably demonstrated that using NIBS methods to stimulate specific brain regions implicated in dyslexia is effective as a therapeutic intervention for specific deficits in individuals with dyslexia, as well as in aiding reading and language function more broadly. However, notably missing from the literature is stimulation of a key region in the ventral pathway of the reading network, the VWFA, as well as deep brain regions implicated in dyslexia, such as the striatum. This gap is consequent of the shortcomings of conventional methods like (HD-)tDCS and tACS. Namely, the trade-off between focality and depth of penetration that these methods unavoidably suffer from precludes focal investigation of deep brain regions. For example, if the striatum were to be identified as a target for stimulation, high-definition stimulation would simply not penetrate the neocortex deeply enough to reach it. Meanwhile, non-high-definition stimulation methods could potentially stimulate the striatum at the cost of weaker electric fields at the target and stronger stimulation in all surrounding cortical areas, making it difficult to attribute observed behavioral changes to the stimulation of a specific region. In other words, it is extremely challenging, if not impossible, to investigate certain dyslexia-implicated brain regions using conventional stimulation methods. It is for this reason that we turn to tTIS.

### 4.2 Transcranial temporal interference stimulation (tTIS)

Recently, a new method called tTIS has been developed (Grossman et al., 2017). To understand the utility of tTIS, it is helpful to understand the mechanisms of transcranial electrical stimulation methodologies used heretofore.

tDCS involves placing electrodes on the scalp and flowing a direct current from the anode(s) to the cathode(s) through a targeted region in the brain. In anodal stimulation, the current depolarizes neurons, increasing the probability of an action potential, thereby facilitating their activation during a cognitive task; in cathodal stimulation, neurons are hyperpolarized, reducing excitability and making action potentials harder to achieve (Nitsche et al., 2008). In tACS, an alternating current flows between electrodes, modulating the oscillatory activity of neuronal networks by entrainment, whereby neuronal firing rates align with the frequency of stimulation (Antal and Paulus, 2013). tACS can have either facilitatory or inhibitory effects depending on the frequency and relative phase of stimulation as well as stimulation site (Antal and Paulus, 2013). It is also important to note that the biochemical mechanisms involved in tES-induced plasticity remain to be fully elucidated (Fertonani and Miniussi, 2017).

Conventional tDCS and tACS rely on two large electrodes usually spaced far apart. Depending on the placement of electrodes, the stimulation can reach deep brain regions, however, this depth of penetration is accompanied by low focality, leading to stimulation of brain regions outside the region of interest, which additionally leads to greater interindividual variability (Mikkonen et al., 2020). To solve the issue of focality, high-definition tES methods use a single small electrode surrounded by four small electrodes. This configuration allows for precise stimulation; however, it is limited to cortical areas (Thair et al., 2017). Thus, conventional tES and HD-tES methods pose a trade-off: You either have deep stimulation or focal stimulation, but not both. tTIS circumvents this trade-off by using two interfering high-frequency alternating currents.

**Figure 1:**
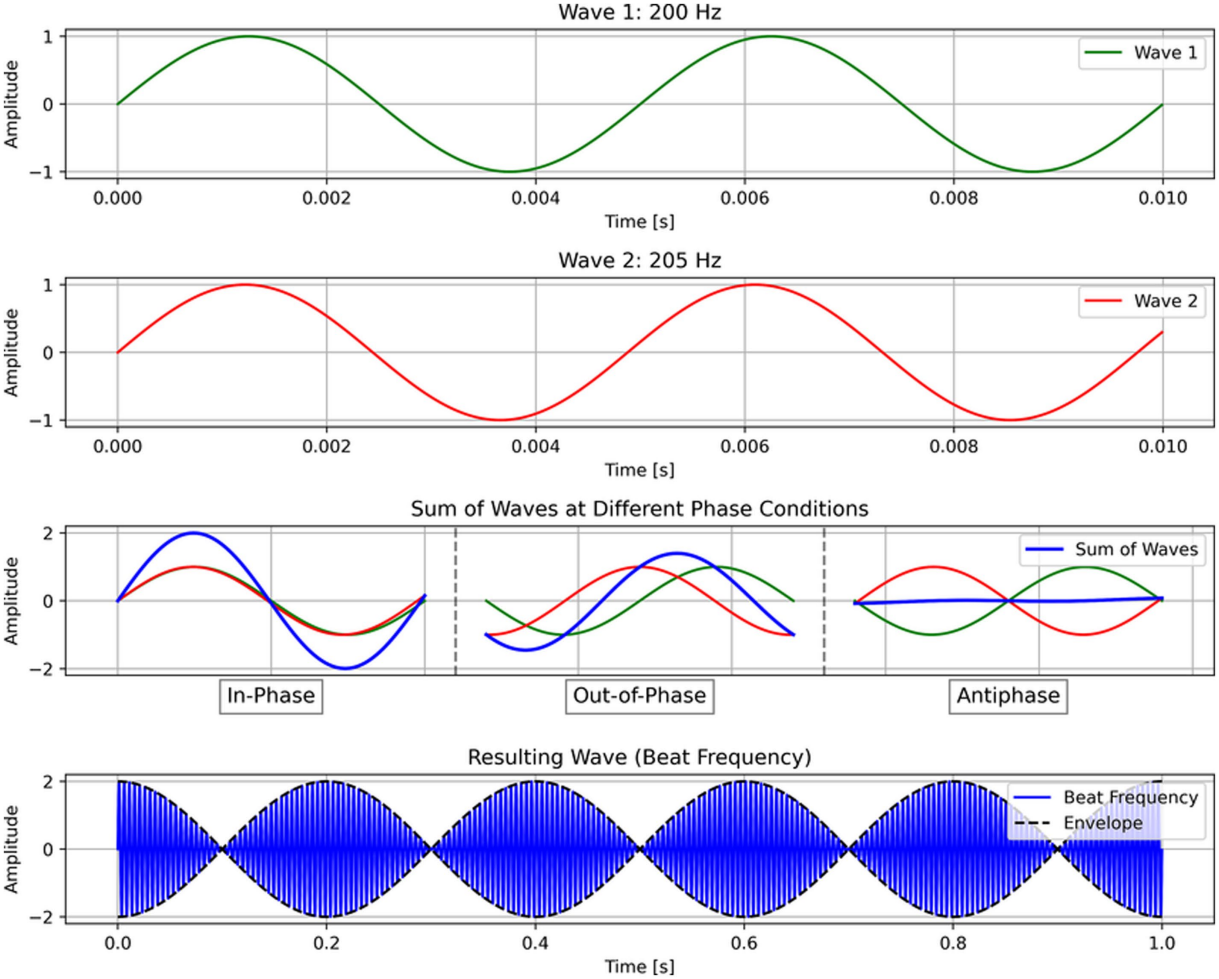
Schema of transcranial temporal interference stimulation (tTIS). In tTIS, high-frequency alternative currents interfere to create a beat frequency (Δf). For illustration purposes, this example shows 200 and 205 Hz, although in practice, tTIS should utilize much higher frequencies (e.g., 2,000 Hz).

tTIS relies on two key principles: First, high-frequency stimulation (> 1 kHz) has no effect on neural activity; second, overlapping electric fields of alternating currents interfere to create a beat frequency (Figure 1) at the difference of the two frequencies (Grossman et al., 2017). Therefore, the two electrode pairs and stimulation frequencies can be optimally chosen such that their electric fields overlap and interfere at any single given region in the brain without effectively stimulating surrounding regions. In this way, tTIS can achieve both focal and deep brain stimulation.

**Figure 2:**
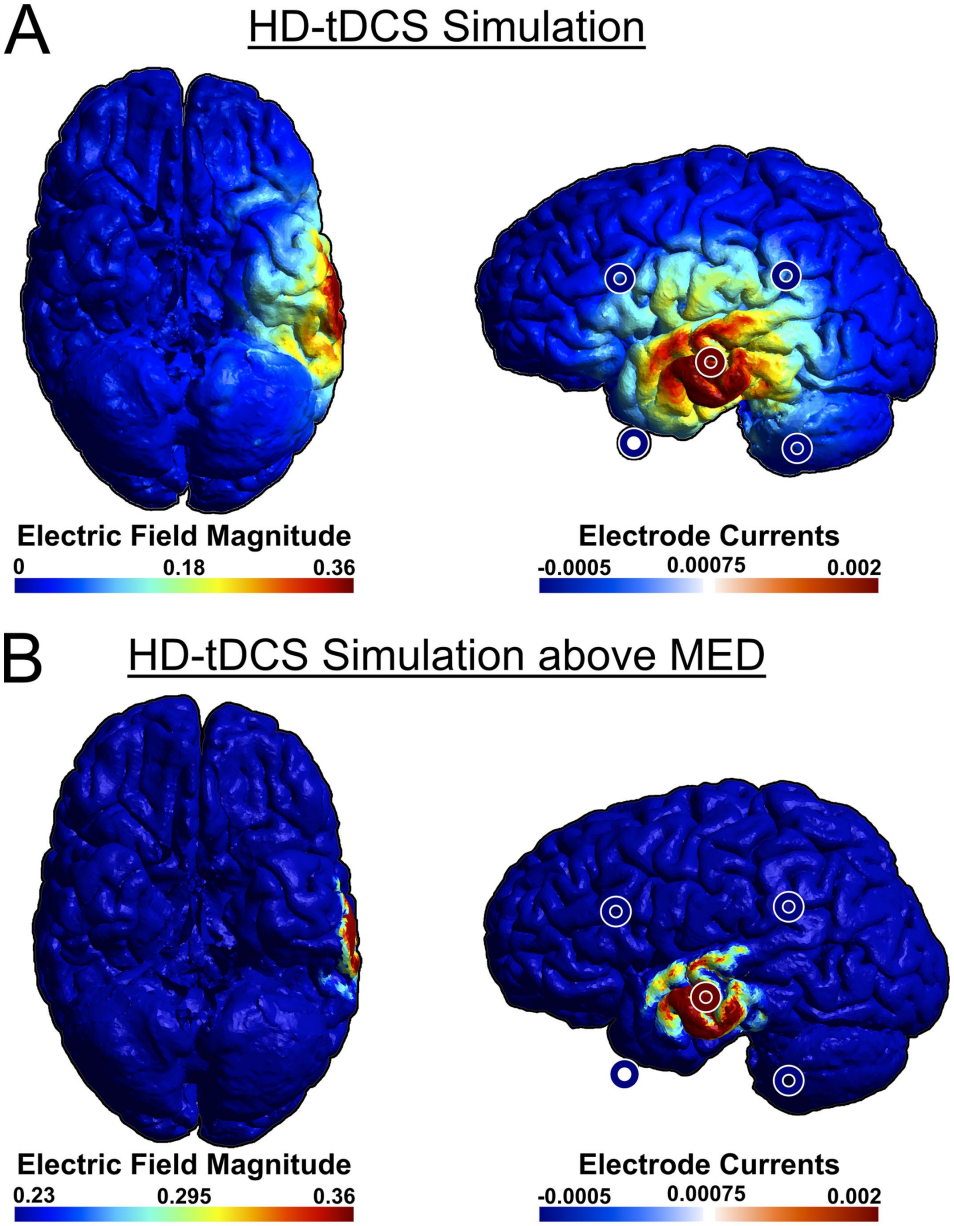
Anodal simulation of HD-tDCS using a 4 × 1 ring electrode montage attempting to stimulate VWFA from the lowest available electrode positions. We used a central anode at T7, with cathodes at FT9, TP9, FC5, and CP5. SimNIBS software was used for the simulation (Thielscher et al., 2015). For ease of visualization, stimulation electrodes were overlaid on the original image. **(A)** HD-tDCS simulation, **(B)** HD-tDCS simulation with adjusted color scale to show field magnitudes exceeding the minimum effective dose (MED). Because the scalp is not depicted in the rendering, the electrodes may appear to be floating due to perspective distortion when projecting a 3D image onto a 2D plane. This distortion affects the perceived distance between the electrodes and the brain surface.

**Figure 3:**
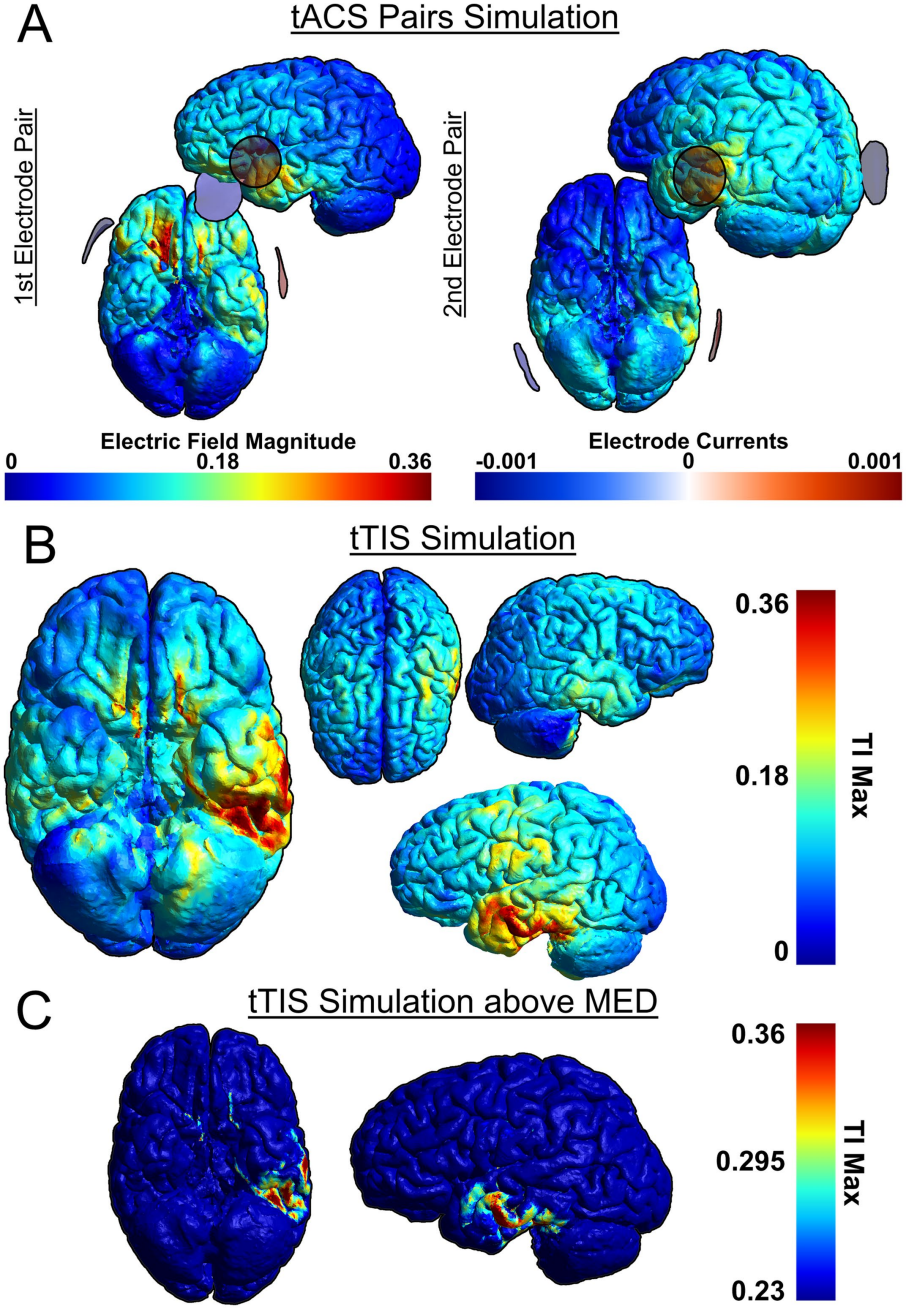
Simulation of tTIS on the VWFA, based on two pairs: FT7/F10 and TP7/P10. SimNIBS software was used for the simulation (Thielscher et al., 2015). **(A)** Individual tACS pairs used for tTIS, **(B)** Combined tTIS field, **(C)** Combined tTIS field with adjusted color scale to show field magnitudes exceeding the minimum effective dose (MED). Because the scalp is not depicted in the rendering, the electrodes may appear to be floating due to perspective distortion when projecting a 3D image onto a 2D plane. This distortion affects the perceived distance between the electrodes and the brain surface.

As a recent development, tTIS has not yet been used in many studies investigating cognitive functions, including language. However, in one study, theta-burst tTIS of the striatum was shown to enhance motor learning in older healthy individuals (Wessel et al., 2023), which demonstrates the efficacy of tTIS to selectively stimulate deep-brain structures as well as the viability of using tTIS as a therapeutic intervention. To compare the efficacy of stimulation, we have simulated attempting to stimulate the VWFA with both HD-tDCS (Figure 2) and tTIS (Figure 3) using SimNIBS software (version 4.1.0) (Thielscher et al., 2015). For the HD-tDCS simulation, the closest possible electrodes to the VWFA were chosen. For the tTIS simulation, we computed the optimal electrode combination with the TI Planning (TIP) tool by IT’IS software (IT’IS Foundation, Zurich, Switzerland; https://itis.swiss). We manually selected a subset of all electrodes based on geometry of the brain and the target region, at which point the software exhaustively tested all possible combinations therein and determined that the optimal electrode combinations were FT7/F10 and TP7/P10.

It can be seen that HD-tDCS fails to effectively stimulate the VWFA, which is difficult to reach on the ventral side of the occipito-temporal cortex, whereas tTIS can stimulate the VWFA much more effectively. It can also be seen in Figures 2A, 3B that tTIS has a broader cortical spread compared to HD-tDCS. However, a 2022 meta-analysis by Alekseichuk et al. showed that the mean minimum effective dose for tACS in awake mammals is 0.23 mV/mm (Alekseichuk et al., 2022). In light of this, most of the observed peripheral stimulation in 3B does not meet this threshold and will therefore not affect cortical activity (Figure 3C, see also Figure 2B). Still, this limitation on focality can be mitigated even further by the use of multi-channel tTIS, whereby more than one pair is used (Song et al., 2021). On the other hand, in case of more superficial target regions, it is clear that HD-tDCS or HD-tACS is the simpler option. Thus, it is necessary to choose the appropriate stimulation method for the target.

Although the mechanism of action of tTIS remains to be fully understood and it seems plausible to use tTIS for either downregulation or upregulation depending on stimulation parameters such as beat frequency, one study showed that tTIS downregulated neural activity (Carmona-Barrón et al., 2023), while previous stimulation studies failed to show facilitatory effects in already proficient readers (Cancer and Antonietti, 2018). Taking that together, we contend that it is more prudent to first attempt to use tTIS to elucidate the neurological pathogenesis of dyslexia subtypes and create a model for dyslexia subtypes in neurotypical adults. Thereafter, its use as a novel therapeutic intervention for individuals with dyslexia can be investigated with a more comprehensive theoretical understanding.

Thus, by stimulating the brain regions revealed in a functional and structural clustering analysis and observing the behavioral effects of downregulation in those regions, we can infer relationships (whether they are ultimately causal or probabilistic) between neuropathologies and specific behavioral deficits of dyslexia. For example, if a clustering analysis reveals functional or structural anomalies in the VWFA, we can use tTIS to stimulate the VWFA and subsequently check for deficits in PA, RAN, etc. To ensure that any observed effects are specifically due to brain stimulation rather than placebo effects or unrelated variability, a sham stimulation group will serve as a control. Additionally, because dyslexia subtypes involve diverse cognitive deficits, it is crucial to test for a broad range of dyslexia-related symptoms rather than assuming *a priori* a one-to-one correspondence between the stimulated region and a single deficit. Furthermore, in light of the neural noise hypothesis, which postulates that variability across the reading network gives rise to dyslexia, tTIS enables us to artificially modulate activity in implicated regions and directly test whether such neural noise mechanisms are causal to dyslexic symptoms. In this way, we can reveal the causal neuropathologies of dyslexia subtypes and temporarily induce a specific subtype of dyslexia in neurotypical individuals for the purpose of investigating the efficacy of different therapeutic interventions.

As with all NIBS methods, inter-individual variability is an important consideration for tTIS. Factors such as skull thickness, cortical folding, and baseline functional connectivity can all shape the distribution of electric fields in the brain. At the group level, this limitation can be overcome with a sufficiently large participant pool, in which case the inter-individual differences average out. On the individual level, the more comprehensive solution is to tailor stimulation to each person’s anatomy. Structural MRI-based simulation software, such as SimNIBS, allows for individualized electric field simulations, enabling the selection of optimal electrode configurations for each participant’s brain structure (Thielscher et al., 2015). The issue of variability in susceptibility to stimulation is less controllable but can be alleviated at the group level with statistical techniques that account for the randomness of participants, like linear mixed models.

Finally, it is important to note the safety of tTIS. Although it is a new method, it is mechanistically similar to tACS, thus tACS safety protocols and guidelines can be used as a baseline for considering the risks associated with tTIS. Although adverse effects such as headache, skin rash, fatigue, etc., are certainly possible, when following standard protocols, the likelihood of adverse effects is small, and their severity is generally mild (Antal et al., 2017; Matsumoto and Ugawa, 2017). Antal et al. further distinguish between controllable and uncontrollable parameters, with the former category comprising stimulation parameters such as electrode montage, amplitude, frequency, etc., and the latter category comprising factors like individual tissue properties, gender, age, baseline state of the brain, etc. (Antal et al., 2017). Due to the highly individual nature of these uncontrollable parameters, safety cannot be reduced to simply setting appropriate stimulation parameters. Nevertheless, adverse effects of tACS are typically limited to mild skin pain and headaches, both of which are transient and avoidable by adjusting stimulation intensities and durations (Antal et al., 2017). Importantly, neurophysiological effects on oscillatory activity induced by tACS are temporary (Kasten et al., 2019), thus any induced dyslexic symptoms will dissipate within a few hours.

While tACS is widely regarded as safe, tTIS differs in its ability to reach deeper regions of the brain. Although direct investigations into the safety of tTIS are still limited, a recent study involving 119 patients analyzed the safety profile of deep brain stimulation, showing that participants rated tTIS-evoked sensations as mild and indistinguishable from placebo (Vassiliadis et al., 2024). They further explain that of their more than 250 sessions, no adverse effects were reported, with the sole exception of a participant for whom the sensation caused the recollection of a prior traumatic brain injury (Vassiliadis et al., 2024). To date, there is no evidence that the stimulation of deep-brain regions introduces any categorical risk beyond those in tACS protocols. These findings present tTIS as a promising tool that remains well within the safety margins of other stimulation protocols.

Taking all of this together, tTIS offers a safe method for downregulating activity at a single specific region anywhere in the brain, which, when applied judiciously, can temporarily create models of dyslexia subtypes in neurotypical adults.

## 5 Discussion

In this hypothesis article, we have proposed a dual-pronged approach for elucidating the neurological pathogenesis of distinct dyslexia subtypes and hastening the development of therapeutic interventions for them. In the first prong, we propose the novel application of PCA and clustering techniques on multimodal brain data to derive neuropathologically distinctive subgroups within the broader group of dyslexic individuals. This will inform the second prong of our approach, which revolves around the novel application of brain stimulation to dyslexia research. By choosing the stimulation technique to suit the target, we can more selectively investigate dyslexia-related regions regardless of their depth in the brain: tTIS allows for focal stimulation of deeper structures, whereas HD-tDCS and HD-tACS effectively stimulate more superficial targets, such as the superior middle gyrus or inferior frontal gyrus. In either case, we will observe the behavioral deficits induced by the stimulation. This will allow the establishment of either causal or probabilistic relationships between the structurally/functionally anomalous brain regions involved in dyslexia and specific dyslexia symptoms, such as PA deficits, RAN deficits, etc. At the same time, by successfully inducing subtype-specific dyslexia symptoms, a model for each subtype can be created in neurotypical adults, which may in turn help aid our understanding of dyslexia from the perspective of single-, double-, or multiple-deficit perspectives while also allowing evaluation of the neural noise hypothesis.

As mentioned, the inability to use animal models that accurately reflect literacy deficits is a loss for dyslexia research. Using neurotypical adult human models for dyslexia would be a helpful substitute to fill in the gap. For example, if we have a reliable protocol for creating a dyslexia model whereby the neurotypical individual is induced with working memory-related perceptual deficits, we can more efficiently investigate and compare the efficacies of various behavioral interventions, such as working memory training protocols. Likewise, with a reliable model recreating PA deficits, we can efficiently assess behavioral interventions intended to help with PA deficits.

Aside from human models of dyslexia, NIBS can be used directly as a remedial tool. As previously mentioned, several studies have already confirmed the efficacy of tDCS, tACS, and TMS in treating dyslexia and related cognitive functions. By using tTIS to upregulate activity in regions of hypoactivation, children with dyslexia can directly benefit and have their symptoms alleviated. And thanks to the established safety of NIBS, it can be more readily deployed in clinical populations. In contrast, pharmaceutical interventions are costly to develop, often expensive for patients, and can take years before testing is finished and a drug makes it to market. Thus, with appropriate stimulation therapies, individuals with dyslexia can receive faster and more affordable treatment than is often available with drugs. In this way, we can open the door to more efficient therapeutic interventions that are precisely tailored to the subtype of dyslexia afflicting the individual seeking treatment.

By no means do we hypothesize this to be a comprehensive solution, since it is inherently limited by its transient induction of symptoms. Indeed, a key limitation in our approach is that we are proposing to temporarily (i.e., short-term) replicate a developmental (i.e., long-term) disorder. In true dyslexia, neuropathologies affect neural development in widespread ways, such as altering functional connectivity across networks, not just disrupting isolated regions, and giving rise to compensatory mechanisms. Regarding functional connectivity, it has been shown that stimulation can also affect oscillatory synchronization with interconnected regions (Liew et al., 2014). In that case, we may be able to more closely replicate true dyslexia, but it would require additional testing to confirm one way or the other. Regarding compensatory mechanisms, it is of course possible to upregulate a region of the brain so as to model dyslexia-caused hyperactivation. However, compared to adults, these regions of hyperactivation are less consistent in children, as these compensatory pathways have not yet been established (Shaywitz and Shaywitz, 2005). Thus, by stimulating healthy adults without dyslexia, we are effectively simulating early childhood dyslexia prior to the development of compensatory mechanisms, though anatomical differences between child and adult brains remain. Depending on the goals of the investigation, this may be desired or may require additional protocols to model compensatory strategies as well, but it is in any case an important consideration for experimental design.

Aside from functional replication of dyslexia, our model is clearly ill-suited for replicating the morphology of dyslexia (subtypes). That is to say, neuromodulation is unable to induce volumetric changes in stimulated brain regions over a single session. This is ultimately a good thing as it would be both a grave violation of ethics and entirely undesirable to permanently alter or impair a healthy participant’s neural anatomy and/or function. Nevertheless, it stands to reason that certain structural abnormalities in dyslexia may not be properly investigable with this approach.

Additionally, the approach outlined herein would not reveal anything of the genetic underpinnings of dyslexia or its subtypes. Thus, this approach is not intended to be a one-size-fits-all kind of approach. However, from a functional perspective, we expect to adequately replicate the functional and connectivity deficits observed in dyslexia. To that end, certain aspects of dyslexia can be investigated more rapidly and more specifically than has been possible so far.

Finally, it is important to note that the model proposed herein is based on healthy adults, whereas dyslexia is a developmental disorder with symptoms that first arise in childhood. Because adult brains differ from child brains, this is, in one sense, a limiting factor of our model. Nevertheless, from a broad neuroscientific perspective, behavioral symptoms induced by transcranial stimulation elucidate causal factors in cognitive function. For that reason, we can still glean important insights into the neural underpinnings of dyslexia and its subtypes, which can help refine early screening tools and guide the development of behavioral interventions. At the same time, we are establishing an experimental protocol that can be applied to various populations, including children. In one study, the effect of tACS on children was shown to be comparable to that of adults in terms of modulating cortical excitability of the motor cortex (Splittgerber et al., 2020). tACS has even been shown to be a more effective treatment for children with attention deficit hyperactivity disorder than Ritalin (Farokhzadi et al., 2020). Thus, with the safety of transcranial stimulation well established, its use in child populations is both ethical and clinically beneficial. Regarding its use to specifically treat dyslexia, as many of the studies referred to herein have shown, stimulation is well-suited as a therapeutic intervention for dyslexia when it is received in tandem with behavioral training. Thus, rather than supplanting existing behavioral interventions, we envision stimulation as a powerful tool to be used alongside them.

In sum, we propose that by combining a clustering approach to structural/functional MRI data with selective downregulating stimulation by tTIS, we can gain a deeper and more fundamental understanding of dyslexia subtypes, we can create subtype-specific dyslexia models in neurotypical adults, and we can ultimately improve the research environment for effective investigation into individually tailored treatment of dyslexia. Additionally, by demonstrating the efficacy of using tTIS to create neurotypical models of dyslexia, it may further prove the viability of employing the same methodological approach for investigating any number of other developmental and psychological disorders. Ultimately, we believe that by opening up this new avenue of research, we can more rapidly help improve the lives of those afflicted with dyslexia.

## Data Availability

The original contributions presented in the study are included in the article/Supplementary material, further inquiries can be directed to the corresponding author. A previous version of this work has been shared as a preprint on bioRxiv (Gallagher et al., 2025).
