# Correction: Modeling dyslexia in neurotypical adults by combining neuroimaging and neuromodulation techniques: a hypothesis paper

**DOI:** 10.3389/fnhum.2025.1732546

**Published:** 2025-11-12

**Authors:** Daniel Gallagher, Zian Huang, Shinri Ohta

**Affiliations:** 1 Department of Linguistics, Faculty of Humanities, Kyushu University, Fukuoka, Japan; 2 Department of Linguistics, Graduate School of Humanities, Kyushu University, Fukuoka, Japan

**Keywords:** dyslexia, dyslexia subtypes, human models, functional magnetic resonance imaging (fMRI), neuropathological clustering, neuromodulation, non-invasive brain stimulation (NIBS), transcranial temporal interference stimulation (tTIS)

In the published article, there was an error in the legend for **Figure 2** as published. The authors reported the wrong electrode combinations. The corrected legend appears below.

**Figure 2**. Anodal simulation of HD-tDCS using a 4 × 1 ring electrode montage attempting to stimulate VWFA from the lowest available electrode positions. We used a central anode at T7, with cathodes at FT9, TP9, FC5, and CP5. SimNIBS software was used for the simulation (Thielscher et al., 2015). For ease of visualization, stimulation electrodes were overlaid on the original image. **(A)** HD-tDCS simulation, **(B)** HD-tDCS simulation with adjusted color scale to show field magnitudes exceeding the minimum effective dose (MED). Because the scalp is not depicted in the rendering, the electrodes may appear to be floating due to perspective distortion when projecting a 3D image onto a 2D plane. This distortion affects the perceived distance between the electrodes and the brain surface.

In the published article, there was an error in the legend for **Figure 3** as published. The authors reported the wrong electrode combinations. The corrected legend appears below.

**Figure 3**. Simulation of tTIS on the VWFA, based on two pairs: FT7/F10 and TP7/P10. SimNIBS software was used for the simulation (Thielscher et al., 2015). **(A)** Individual tACS pairs used for tTIS, **(B)** Combined tTIS field, **(C)** Combined tTIS field with adjusted color scale to show field magnitudes exceeding the minimum effective dose (MED). Because the scalp is not depicted in the rendering, the electrodes may appear to be floating due to perspective distortion when projecting a 3D image onto a 2D plane. This distortion affects the perceived distance between the electrodes and the brain surface.

In the published article, there was an error. The authors reported the wrong electrode combinations.

A correction has been made to **4 Transcranial temporal interference stimulation (tTIS) for modeling dyslexia subtypes**, *4.2 Transcranial temporal interference (tTIS)*, Paragraph 5. This sentence previously stated:

“We manually selected a subset of all electrodes based on geometry of the brain and the target region, at which point the software exhaustively tested all possible combinations therein and determined that the optimal electrode combinations were F7/F10 and T7/P8.”

The corrected sentence appears below:

“We manually selected a subset of all electrodes based on geometry of the brain and the target region, at which point the software exhaustively tested all possible combinations therein and determined that the optimal electrode combinations were FT7/F10 and TP7/P10.”

The original article has been updated.

